# A Case of Clinical and Viral Rebound Following Completion of a Five-Day Course of Ensitrelvir for COVID-19

**DOI:** 10.7759/cureus.107623

**Published:** 2026-04-23

**Authors:** Yu Kasamatsu, Satoshi Shiraisi, Masato Uji, Masanori Kobayashi, Tetsushi Goto, Yoshikazu Hiura

**Affiliations:** 1 Infectious Disease, Osaka City Juso Hospital, Osaka, JPN; 2 Respiratory Medicine, Osaka City Juso Hospital, Osaka, JPN; 3 General Internal Medicine, Osaka City Juso Hospital, Osaka, JPN

**Keywords:** covid-19, covid-19 relapse, ensitrelvir, rebound, viral infection

## Abstract

Ensitrelvir inhibits the 3-chymotrypsin-like protease of severe acute respiratory syndrome coronavirus 2 and accelerates viral clearance and symptom improvement through its potent antiviral activity. Reports of rebound after ensitrelvir treatment are scarce. This report presents a case of a healthcare worker in her 50s who had no significant underlying conditions. As a close contact, she underwent nasopharyngeal polymerase chain reaction (PCR) testing after her sister was diagnosed with coronavirus disease. The test result was positive with a cycle threshold (Ct) value of 31.97 by using Cobas® liat. Ensitrelvir treatment was initiated because of a slight sore throat, and rapid improvement was observed starting the following day. Treatment was completed on day five. Examination on day six indicated that the symptoms had resolved. However, the sore throat recurred on day seven, antigen levels increased again, and fever and cough developed on day eight. On day nine, a Ct value of 16.6 was noted, suggesting a strongly positive result and rebound. She received five vaccine doses and had markedly high levels of anti-S immunoglobulin G (IgG) antibodies, but her anti-N IgG antibodies were negative, indicating that she had a normal immune response and had not been infected with COVID-19. Her illness was mild, and symptoms peaked on day 11, without additional treatment. This case strongly suggests viral rebound and symptom relapse after ensitrelvir administration. Therefore, caution is warranted, as rebound may occur after ensitrelvir treatment, similar to that after nirmatrelvir/ritonavir treatment.

## Introduction

Ensitrelvir is a relatively new antiviral drug that inhibits the 3-chymotrypsin-like protease of severe acute respiratory syndrome coronavirus 2 and accelerates viral clearance and symptom improvement in mainly vaccinated individuals infected with the Omicron variant [1,2].

For some antiviral drugs, such as nirmatrelvir/ritonavir (NIR/rtv), a "rebound phenomenon" has been reported, in which patients test positive by polymerase chain reaction (PCR) or experience a recurrence of symptoms after completing a five-day course of treatment [3-7]. However, it remains unclear whether a rebound phenomenon occurs following treatment with ensitrelvir.

Herein, we report the first case of clinical and viral rebound after ensitrelvir treatment.

## Case presentation

This case involved a woman in her 50s, employed as a physician, with no significant medical history, comorbidities, or history of coronavirus disease (COVID-19). She was a non-smoker and occasional drinker and had no possibility of pregnancy. She had received six doses of the COVID-19 vaccine, all Pfizer, with the last dose administered six months prior.

The patient's sister, who lived with her, had been diagnosed with COVID-19. As a close contact, the patient underwent a nasopharyngeal PCR test, which was positive (cycle threshold (Ct) value: 31.97; Cobas® liat). She had a slightly sore throat and desired early improvement; therefore, ensitrelvir treatment was initiated (day one). The patient's sore throat improved the following day. After completing the five-day course, the symptoms had resolved, as confirmed at a follow-up visit on day six. The quantitative antigen test (Lumipulse®) showed a low value (144.8 pg/mL), and she was scheduled to return to work on day seven.

**Figure 1:**
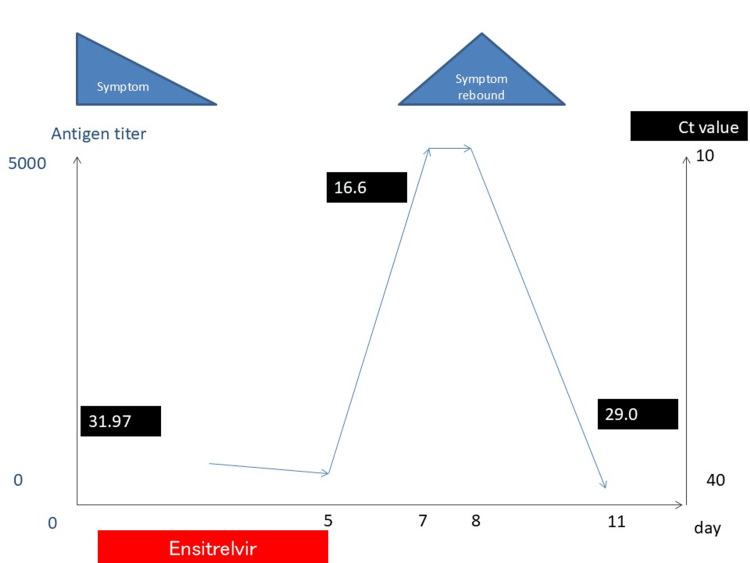
Clinical course Progression of symptoms, antigen levels, and viral load Image created by the authors using PowerPoint 2016 (Microsoft® Corp., Redmond, WA).

However, she presented for re-evaluation on day seven because of the recurrence of a sore throat, along with low-grade fever and cough that began that morning. Her physical findings were as follows: body temperature of 37.9°C; peripheral capillary oxygen saturation of 97%; blood pressure of 114/64 mmHg; pulse rate of 80/min; and mild pharyngeal erythema. Laboratory tests revealed a white blood cell count of 7,000, with differential counts within the normal range. Lactate dehydrogenase level was 171 IU/L, and KL-6 level was 173 U/mL (Table 1). Chest radiography revealed no evidence of pneumonia (Figure 1).

**Table 1:** Laboratory data Patient's laboratory data show a slightly elevated C-reactive protein (CRP) level and COVID-19 antigen titer exceeding the detection limit.

Parameter	Result	Reference Range
WBC	7070	3170-8400 /μL
RBC	389×10^4^	372-506×10^4^/μL
Hb	11.0	11.0-14.7 g/dL
Ht	34.9	35.2-46.7％
MCV	90	87-101 fl
Plt	24.8×10^4^	16.7-39.0 /μL
Neu	70.6	39.7-71.2％
Lym	20.5	21.9-50.3％
Mono	7.9	4.2-9.6％
Eos	0.4	0.6-4.9％
Bas	0.6	0.02-0.07％
LDH	171	124-222 U/L
AST	15	8-38 U/L
ALT	8	4-44 U/L
Na	140	138-145 mEq/L
K	4.1	3.6-4.8 mEq/L
Cl	104	101-108 mEq/L
BUN	11.2	8.0-20.0 mg/dL
Cre	0.49	0.40-0.80 mg/dL
CRP	0.6	<0.26 mg/dL
Glu	109	73-109 mg/dL
HbA1c	5.6	4.6-6.2％
PT	10.3	9.9-11.8 sec
APTT	25	24.0-37.0 sec
D-dimer	0.7	<1.0 μｇ/mL
T-cho	219	150-219 mg/dL
KL-6	173	0-500 U/mL
SARS-COV-2 antigen titer	>5000	<1.0 pg/mL
HBsAg	(-)	(-)
HCVAb	(-)	(-)
HIVAg・Ab	(-)	(-)

**Figure 2:**
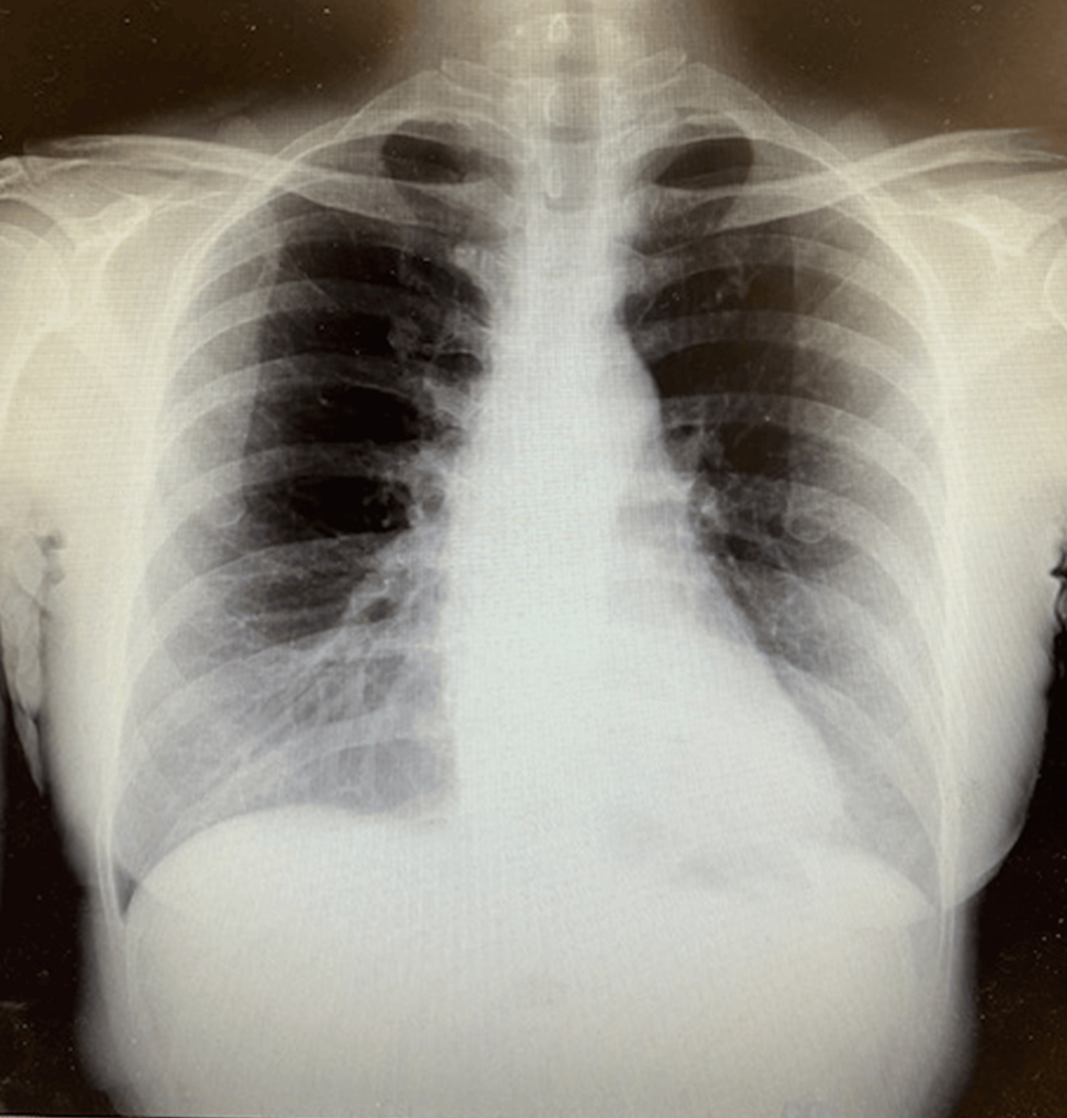
Chest X-ray Chest radiography showed no abnormal shadow.

Although the symptoms had completely resolved after ensitrelvir administration, 48 hours after discontinuation, the sore throat recurred along with fever (37.9°C) and cough onset. Quantitative antigen levels increased significantly from 144.8 to ≥ 5,000, and Ct values rose markedly from 31.97 to 16.6 (Figure 2). This case strongly suggests viral rebound and symptom relapse after ensitrelvir administration. The patient showed no new signs of pneumonia and only mild symptoms that resolved spontaneously within a few days without additional treatment. She had no history or comorbidities suggesting immunodeficiency and tested negative for HIV, with high CD4 counts and anti-S IgG antibodies, indicating no immune compromise. Her anti-N IgG antibodies were also negative, confirming that this was the first infection.

## Discussion

Ensitrelvir is a relatively new antiviral drug for COVID-19, and this is the first case report of clinical and viral rebound. In the Phase 3 SCORPIO-SR trial, transient viral load increases were reported after dosing cessation (ensitrelvir 7.8% vs. placebo 4.7%); however, no reports of viral rebound accompanied by clinical rebound, as observed in this case, were documented [1]. Encitrelvir has a longer half-life (44-54 hours) than NIR/rtv (3.4-4.8 hours), suggesting that it is less prone to allowing a rebound [8]. Therefore, our case, featuring clinical rebound accompanied by viral rebound, is valuable.

For NIR/rtv, a drug in the same class, the EPIC-HR trial reported a rebound rate of 5.2% (3.8% in the placebo group) and symptom recurrence rate of 12.2% (16.1% in the placebo group) [3]. Another prospective observational study reported a symptom recurrence rate of 18.9% (24/127 cases) for NIR/rtv versus 7.0% (3/43 cases) for the control group (p = 0.06) [4]. Risk factors identified by Smith et al. included immunodeficiency (7.37), age between 18 and 65 years (3.09), Charlson comorbidity index > 6 (6.02), and steroid use (7.51) [6]. Camp et al. described a black female patient with underlying comorbidities and no prior infection [7]. Our case had sufficient antibody titres post vaccination and no comorbidities; however, certain patient characteristics, such as age, female sex, and lack of prior infection, correspond to previously reported rebound risks. Further studies are required to determine the frequency of rebound with encitrelvir and associated risk factors.

## Conclusions

In conclusion, we report a case that strongly suggests viral rebound and symptom relapse after ensitrelvir administration. Ensitrelvir is a relatively new antiviral drug for COVID-19, and this is the first reported case of clinical and viral rebound. Therefore, caution is warranted, as rebound may occur after ensitrelvir treatment, similar to that after NIR/rtv treatment. Although rebound of ensitrelvir was thought to be unlikely due to the drug's long plasma half-life, further study on rebound cases is needed.
